# A Correlation Between the Pathogenic Processes of Fibromyalgia and Irritable Bowel Syndrome in the Middle-Aged Population: A Systematic Review

**DOI:** 10.7759/cureus.29923

**Published:** 2022-10-04

**Authors:** Carla Valencia, Hameeda Fatima, Ijeoma Nwankwo, Mahvish Anam, Shrinkhala Maharjan, Zainab Amjad, Abdelrahman Abaza, Advait M Vasavada, Akhil Sadhu, Safeera Khan

**Affiliations:** 1 Internal Medicine, California Institute of Behavioral Neurosciences & Psychology, Fairfield, USA

**Keywords:** fibromyalgia syndrome, fibromyositis, mucous colitis, pathogenesis, fibromyalgia, irritable bowel syndrome

## Abstract

Irritable bowel syndrome (IBS) is a common pathology in middle-aged patients and a regular consultation in the gastroenterology office. The prevalence is high in females with a ratio of 2:1, and due to its multifactorial etiology, it is difficult to address the symptomatology. On the other hand, fibromyalgia syndrome (FMS) is a chronic widespread pain syndrome also prevalent in the female population, characterized by systemic symptoms. It is proven that 28-59 % of patients with FMS develop IBS at some point in their illness; on the other hand, 32-77% of those with IBS will develop FMS. Our study aims to compile information about the pathogenesis of these diseases and highlight their common processes to target these two illnesses potentially.

This systematic review comprises twenty-three studies published between 2017 and 2022, selected by electronic research with keywords and Medical Subject Headings (MESH) strategy. The articles were taken from PubMed, Pubmed Central (PMC), Medline, and Cochrane libraries and met the inclusion and exclusion criteria and the pertinent quality checklists. Of the reviewed studies, 10 were case-control, six were narrative reviews, three were systematic reviews, three were cross-sectional, and one was a cohort study. They investigated the correlation and similitudes in the pathogenic process between FMS and IBS.

There are some similar mechanisms in the physiopathologies of IBS and FMS, where the immune system, especially the mast cells (MCs), along with their products, receptors, the inflammatory cells with their intermediaries, hormones, and neurotransmitters such as serotonin, act together pathologically. Also, the role of the microbiota is very important in this pathogenesis since dysbiosis alters the levels of serotonin in the body and can produce hyperstimulation of the autonomic nervous system.

There are common associated factors in IBS and FMS, with evident symptoms presented in both syndromes such as fatigue, pain, hypersensitivity, depression, anxiety, and others, that could be correlated in a certain way. After this systematic review, we can conclude that the most accepted theories of the common pathogenesis are the role of serotonin and MCs with their inflammatory biomarkers, which can affect different parts of the body producing the characteristic symptomatology. Moreover, other pathogenic mechanisms such as the involvement of microbiota and dysregulation of the gut-brain axis have shown promising results, and further investigation should be made to support their role.

## Introduction and background

Irritable bowel syndrome (IBS) is a common medical consultation in gastroenterology. It is estimated that this syndrome can appear in up to 15% of the general population in developed countries, with a predominance in women in a 2:1 ratio. Most patients are middle-aged, with more than 20% presenting with symptoms of cramping, bloating, recurrent abdominal pain, and changes in bowel movements (consistency or frequency of the stools) without an evident organic origin. There are many studies about probable pathogenesis; however, this is not understood because it is unclear how immune system cells, inflammatory mediators, and other neurochemicals contribute to the development of IBS [1-3]. 

It is important to mention the complexity of this disease due to the influence of several factors such as chronic stress, bowel dysmotility, mucosal inflammation, immune system activation (mast cells), visceral hypersensitivity, neuroendocrine intermediaries, gut flora malfunction, and others. Due to this complexity of pathogenesis, it usually takes longer to diagnose this condition. The patients struggle to adhere to their treatment regimen and may develop other comorbidities. As a common disease with a global prevalence of 10-15%, its diagnosis is made by Rome IV criteria, and this classifies the IBS into four subtypes such as IBS with predominant constipation (IBS-C), IBS with predominant diarrhea (IBS-D), IBS with mixed bowel habits (IBS-M), and IBS unsubtyped or unclassified (IBS-U) [4].

Fibromyalgia syndrome (FMS) is a chronic musculoskeletal widespread pain syndrome that appears in 2% to 8% of the population, most of the time associated with other symptoms such as fatigue, depression, sleep disturbances, decreased cognition, gastrointestinal problems, and poor physical performance with a duration of at least three months [5,6]. 

The etiology of this syndrome is still under research; the most common hypotheses are small fiber neuropathy, central sensitization, and stress-related dysautonomia with neurogenic features. Nevertheless, there are multiple new pieces of information that have proven that various triggers can provoke or worsen the pain. These studies stated that pain pathway impairment, immune cell activation, abnormal signaling in the central nervous system, inflammatory alterations, and sensory hyper-responsiveness affect the outcome of patients with FMS. Moreover, its interaction with other factors such as genetics, environmental, or psychological has an important part in the pathogenesis and development of FMS [6,7]. 

It is interesting to highlight the similarities between syndromes, where the inflammatory pathway and immune system have an abnormal performance and interact with other substances, receptors, and neurologic pathways. Therefore, the coexistence of IBS in FMS patients is a common finding in rheumatology consultation in up to 18% [8].

For all the described above, it is important to continue studying the pathogenesis of these syndromes and be aware of their presence simultaneously or later in the course of these two diseases. In that way, we can anticipate the co-occurrence of IBS and FMS in the middle-aged population and help the patients have a better quality of life. That is why we reviewed and compared some of the new pathogenic discoveries in this field on this research. 

## Review

Methods

This systematic review and its results followed the updated Preferred Reporting Items for Systematic Reviews and Meta-Analyses (PRISMA) guidelines. The research papers selected were important to reach the research question: 'What is the correlation in the pathogenesis of fibromyalgia syndrome and irritable bowel syndrome in the middle-aged population?'. The data extracted was downloaded into EndNote citation software and then saved in an Excel data sheet where the classification, selection, and screening took place depending on the type of study, year, and relevance [9].

Search strategy

We used research databases and search engines such as PubMed, Pubmed Central (PMC), Medline, and Cochrane library to approach the strategy. We searched using keywords and Medical Subject Headings (MeSH) to find the most relevant articles related to the topic of this systematic review [10-12].

In PubMed, PMC, and Medline, we used the MESH strategy for the first disease we obtained: ("Fibromyalgia/diagnosis" [Majr] OR "Fibromyalgia/epidemiology" [Majr] OR "Fibromyalgia/etiology" [Majr] OR "Fibromyalgia/genetics" [Majr] OR "Fibromyalgia/pathology" [Majr] OR "Fibromyalgia/physiology" [Majr] OR "Fibromyalgia/physiopathology" [Majr]); for the second disease it was: ("Irritable Bowel Syndrome/diagnosis" [Majr] OR "Irritable Bowel Syndrome/epidemiology" [Majr] OR "Irritable Bowel Syndrome/etiology" [Majr] OR "Irritable Bowel Syndrome/genetics" [Majr] OR "Irritable Bowel Syndrome/pathology" [Majr] OR "Irritable Bowel Syndrome/physiology" [Majr] OR "Irritable Bowel Syndrome/physiopathology" [Majr] ).

Combined, the strategy was: ("Fibromyalgia/congenital"[Majr] OR "Fibromyalgia/diagnosis"[Majr] OR "Fibromyalgia/epidemiology"[Majr] OR "Fibromyalgia/etiology"[Majr] OR "Fibromyalgia/genetics"[Majr] OR "Fibromyalgia/pathology"[Majr] OR "Fibromyalgia/physiology"[Majr] OR "Fibromyalgia/physiopathology"[Majr] ) AND ( "Irritable Bowel Syndrome/diagnosis"[Majr] OR "Irritable Bowel Syndrome/epidemiology"[Majr] OR "Irritable Bowel Syndrome/etiology"[Majr] OR "Irritable Bowel Syndrome/genetics"[Majr] OR "Irritable Bowel Syndrome/pathology"[Majr] OR "Irritable Bowel Syndrome/physiology"[Majr] OR "Irritable Bowel Syndrome/physiopathology"[Majr] ). 

The keywords used in the Cochrane library were: "Fibromyalgia," "fibromyositis," "Diffuse Myofascial Pain Syndrome," "irritable bowel syndrome," and "Mucous colitis." We obtained the most pertinent research papers, and we used them in different arrangements with the Booleans "AND," "OR." 

Inclusion and exclusion criteria

To meet the inclusion criteria, we selected full-text systematic reviews, observational studies, and literature reviews in the past five years, written in English and peer-reviewed. We extracted relevant papers from the middle-aged population and related them to the topic. We excluded gray literature, unpublished research, pediatric population, elderly population, and studies practiced on animals. The inclusion and exclusion criteria are summarized in Table 1. 

**Table 1:** Description of inclusion and exclusion criteria

INCLUSION CRITERIA	EXCLUSION CRITERIA
Published papers from the past five years	Papers written before the past five years
Papers written in English	Papers written in other languages than English
Papers including middle-aged population	Papers including the pediatric and elderly population
Research papers done in humans	Research papers done in animals
Papers related to the research question	Papers not related to the topic and unpublished

Quality appraisal of studies

We checked the quality of the selected studies with the New Castle Ottawa scale, the scale for the quality assessment of narrative review articles (SANRA) checklist, and the assessment of multiple systematic reviews (AMSTAR) tool for observational, traditional, and systematic reviews, respectively. We obtained an average quality of more than 80%. Thus the 23 papers were approved with very good quality (medium to high). We describe the quality appraisal for studies included in this systematic review in Tables 2-3 [13-15].

**Table 2:** New Castle Ottawa scale assessment tool in observational studies

	Bednarska et al. [2]	Xu et al. [3]	Yang et al. [4]	Khamisy-Farah et al. [5]	Dlugosz et al. [16]	Patel et al. [17]	İliaz et al. [18]	Liu et al. [19]	Zhao et al. [20]	Meira de-Faria et al. [21]	Ivashkin et al. [22]	Ellergezen et al. [23]	Norlin et al. [24]	Chojnacki et al. [25]
Year	2017	2017	2017	2021	2017	2017	2018	2018	2019	2020	2021	2021	2021	2018
Representativeness of the exposed cohort	1	1	1	1	1	1	1	1	1	1	1	1	1	1
Selection of the non-exposed cohort	1	1	1	1	1	1	1	1	1	1	1	1	1	1
Ascertainment of exposure	1	1	1	1	1	1	1	1	1	1	1	1	1	1
Demonstration that outcome of interest was not present at start of study	1	1	1	1	1	1	1	1	1	1	1	1	1	1
Comparability of cohorts on the basis of the design or analysis	0	1	1	1	1	1	1	1	1	1	1	1	1	1
Assessment of outcome	1	1	1	1	1	1	1	1	1	1	1	1	1	1
Was follow-up long enough for outcomes to occur	1	1	1	1	0	0	1	1	1	1	1	1	0	0
Adequacy of follow up of cohorts	0	1	0	1	1	1	1	1	1	1	0	1	0	1
Total	6/8	8/8	7/8	8/8	7/8	7/8	8/8	8/8	8/8	8/8	7/8	8/8	6/8	7/8
Quality of study	Medium	High	High	High	High	High	High	High	High	High	High	High	medium	High

**Table 3:** A Measurement Tool to Assess systematic Reviews (AMSTAR) was used in the included systematic review studies to check their quality RoB=Risk of Bias; PICO=population, intervention, control, and outcomes

AMSTAR Yes/Partial yes/No	Erdrich et al., 2020 [8]	Krammer et al., 2019 [26]	O’Mahony et al., 2021 [27]
Did the research questions and inclusion criteria for the review include PICO components?	Yes	Yes	Yes
Did the report explicitly state that the review methods were established before the review, and did the report justify any significant deviations from the protocol?	Yes	Yes	Yes
Did the review authors explain their selection of the study designs for inclusion in the review?	Yes	Yes	Yes
Did the review authors use a comprehensive literature search strategy?	Yes	Yes	Yes
Did the review authors perform study selection in duplicate?	Yes	Yes	Yes
Did the review authors perform data extraction in duplicate?	Yes	Yes	Yes
Did the review authors provide a list of excluded studies and justify the exclusions?	Yes	Yes	Yes
Did the review authors describe the included studies in adequate detail?	Yes	Yes	Yes
Did the review authors use a good technique for assessing the RoB in individual studies included in the review?	Yes	Yes	Yes
Did the review authors report on the funding sources for the studies included in the review?	Yes	Yes	Yes
If meta-analysis was performed, did the review authors use appropriate methods for the statistical combination of results?	Yes	Yes	Yes
If a meta-analysis was performed, did the review authors assess the potential impact of RoB in individual studies on the results of the meta-analysis or other evidence synthesis?	Yes	Yes	Yes
Did the review authors account for RoB in individual studies when interpreting/discussing the review results?	Yes	No	Yes
Did the review authors provide a satisfactory explanation for and discuss any heterogeneity observed in the results?	Yes	Yes	Yes
If they performed quantitative synthesis, did the review authors conduct an adequate investigation of publication bias (small study bias) and discuss its likely impact on the review results?	Yes	No	Yes
Did the review authors report any potential sources of conflict of interest, including any funding they received for conducting the review?	Yes	Yes	No
Total score	16/16 (high)	14/16 (high)	15/16 (high)

Data extraction

Two investigators extracted data in separate ways. Each investigator selected and reviewed the data based on relevance and following the eligibility criteria. If there were an inconclusive article, both would review the same paper and decide. Then the data was downloaded into EndNote citation software. 

Results

**Figure 1:**
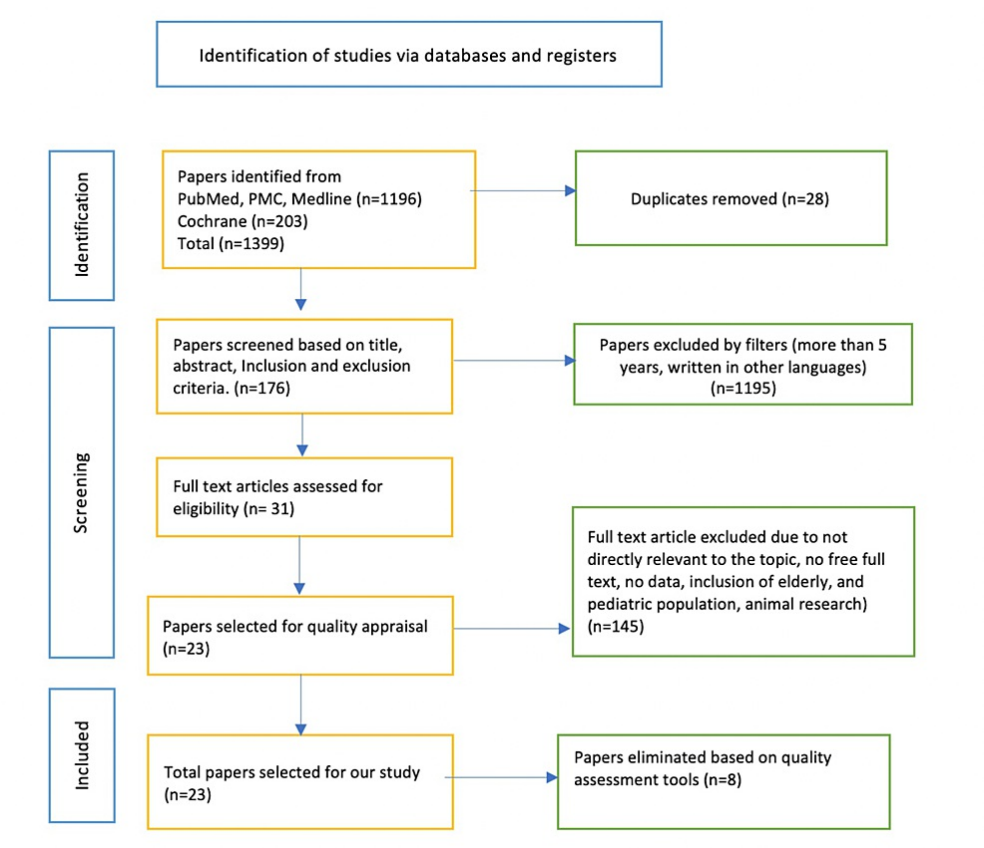
Preferred Reporting Items for Systematic reviews and Meta-Analyses (PRISMA) flow chart PMC=PubMed Central

The total number of articles searched was 1399; 1196 were found in PubMed, PubMed Central (PMC), and Medline, and 203 were found in the Cochrane library. Out of them, 1195 were eliminated because they did not meet the eligibility criteria, and 28 were removed due to duplication. The next step accomplished was the screening of 176 remained articles based on title, abstract, and specific inclusion and exclusion criteria. The final number of articles was 23, which had relevance and met all the appropriateness points for our research. In the end, the quality assessment tools were used to evaluate every paper, with an average quality of more than 80%, considering them moderate to high-quality papers. The details mentioned above are described in the following PRISMA flow chart in Figure 1 [28].

It is important to mention that of the 23 studies selected, there were 10 case-control, six narrative reviews, three systematic reviews, three cross-sectional, and one cohort study. The primary outcomes of these studies were the explanation of the pathogenesis in FMS and IBS addressed by different parameters, including the immune system, especially the role of mast cells (MCs), the inflammatory pathway, cytokines, growth factors, neural-immune-endocrine participation, serotonin activation in nerve fibers, and stimulation of some receptors.

The population included in the observational studies comprised middle-aged males and females (n=34351) with a diagnosis of FMS (n=33912) and IBS (n=439), and control groups (n=135321) with the same characteristics according to each study. In most of them, the number of patients of both diseases was females with 54.7% vs. males with 45.3% for FMS and 56.7% vs. 43.3% for IBS, with a mean age of 50.2 years and 34.6 years, respectively. On the systematic reviews and meta-analyses [8,25,26] results, we had a total of 101 studies with a total number of 7476 patients with the diagnosis of FMS (n=5570) and IBS (n=1906).

Discussion 

IBS and FMS are complex syndromes common in clinical practice and could be present separately or simultaneously. The percentage of patients with FMS that develop IBS is 28-59%, and 32-77% of patients with the diagnosis of IBS develop fibromyalgia during their illness, with a high risk of 1.54-fold. These results suggest a bi-directional correlation between them [4,8].

In the last years, multiple studies have been presented regarding the pathogenesis and physiopathology of FMS and IBS individually. However, few associate the link between both diseases. It is important to know that there are common processes and interesting new points to keep in mind about these syndromes. This could be used as baseline information for the development of new research projects. In consequence, they might generate new therapies to treat or decrease the severity of these illnesses. 

Immune System and Inflammation Intermediaries

The inflammatory pathways with their cells and mediators have an important role in the development of FMS and IBS. As a common process between these two diseases, it is crucial to mention that the MCs are the first immune cells activated in the pathogenesis. Producing pro-inflammatory cytokines such as Interleukins (ILs), IL-1 beta, IL8, IL6, IL17, and tumor necrosis factor-alpha (TNF-α), produce a response in the intestinal mucosa in IBS patients. Their cumulative effect could develop disseminated body pain in FMS. This is corroborated by Ellergezen et al., where there was a positive correlation between the increased levels of IL-17 and the feeling of waking up unrefreshed in the morning in patients with FMS. The authors also asseverate a disruption in the equilibrium of anti-inflammatory and pro-inflammatory processes as part of the pathogenesis of these syndromes [5,17,23].

According to Norlin et al., IBS patients had higher levels of TNF-α (p = 0.001), which were positively correlated with having fatigue, one of the most important common symptoms in IBS and FMS, which impacts the daily life of these patients significantly (rho = 0.25, p = 0.02) [24]. As part of the inflammatory process, cytokines are proteins made by certain cells, such as eosinophils and especially MCs, which influence the activation of the immune system. Various types of cytokines, such as chemokines, interleukins, interferons, leptin, and others, interact with their receptors in the tissues. Their dysregulation is important in the pathogenesis of IBS and FMS [26,29].

Liu et al. demonstrated the significantly increased expression of colonic leptin, a cytokine product of MCs that acts in the inflammatory process, especially in patients with diarrhea predominance IBS (IBS-D) (IBS-D: median, 4424.71; IQR, 4533.63; control: median, 933.65; IQR, 888.10; P < 0.001). It was shown that leptin affected the nerve fibers and positively correlated with the development of anxiety, depression, and disease severity in IBS-D [19]. 

In FMS, the chemokines ligands C, CC, CXC, and CX3C can interact pathologically and overproduce immune cells and their mediators. This can cause inflammation and pain by activating its receptor C-C chemokine receptor type 2 (CCR2), producing membrane depolarization, sensitizing nociceptors, and opening certain channels in the cell membranes. Zhao et al. proposed that the monocyte chemotactic protein 1 (MCP1) or chemokine C-C motif ligand 2 (CCL2) is an important chemokine essential for the recruitment of immune cells that can produce chronic inflammation and be part of the pathogenesis. They also corroborated that there are high levels of this MCP1/CCL2 in patients with FMS, and consequently there is a significant relationship with the severity of the FMS symptoms [20]. 

The literature reviewed about the presentation of the pain in patients with fibromyalgia stands that it may be a consequence of an inflammatory response in the hypothalamus and that there it has found increased levels of IL-8 and other interleukins in blood samples and spinal fluid in patients with FMS. Another cause of the pain is the presence of high levels of corticotropin-releasing hormone (CRH) released by the hypothalamic-pituitary-adrenal axis (HPA) in stress-related situations. This produces the activation of innate immune cells, especially MCs, and causes sensitization of nociceptors and corticotropin-releasing hormone receptors (CRHRs) that increase the expression of the cytokines mentioned above [20,30]. 

Although it is proven that the components of the innate immune response have a critical role in the symptomatology of FMS, the information about the role of T cells in this syndrome is limited. Banfi et al. collected several studies where cluster of differentiation 4+ T cells (CD4+) function was altered but not very revelatory about the adaptive immune system response. Yet, their results were not conclusive, and further investigation is needed. Also, they mentioned that an exaggerated autoimmune reaction could happen at any point of the disease, affect the central pain pathways, and activate some inflammatory mediators in response to factors such as stress [6,18,27]. 

In correlation with the information provided above, there are other studies about the role of IL in the pathogenesis and symptomatology of IBS. Ivashkin et al. concluded that there is an increased expression of IL-1, IL-2, and TNF-α (5.6 ± 0.5 and 1.2 ± 0.3; p < 0.0001) in these patients compared with their controls [22]. These mediators impaired the intestinal barrier, inhibited the secretion of neurotransmitters such as serotonin and dopamine in the gut and mesolimbic cortex, and maintained a chronic low-grade inflammation producing the typical symptomatology of IBS [22,24]. 

This could be related to the conclusion of Khamisy-Farah et al., where the authors described the presence of significantly high levels of platelet-lymphocyte ratio (PLR) (143.6 vs. 107.6, P < 0.001) and C-reactive protein (CRP) in the serum of patients with the diagnosis of FMS as biomarkers of inflammatory processes. These substances might be useful for considering the FMS diagnosis when there is no notorious organic cause [5].

To group all this information, we can say that IBS and FMS might not have common processes working in the same location. Still, the inflammatory mediators and immune cells involved in both pathologic processes act systemically, and their products are part of their symptomatology. Thus, concurrent inhibition or control of such inflammatory cells and intermediaries may significantly contribute to simultaneously reducing the symptoms and severity of these syndromes. We summarize the information related to this subheading in Table 4. 

**Table 4:** Immune and inflammatory mechanisms correlated with IBS and FMS pathogenesis IBS=Irritable Bowel Syndrome; FMS=Fibromyalgia syndrome; CRP=C-reactive protein; PLR=Platelet-lymphocyte ratio; HC=Healthy controls; FGIDs=Functional gastrointestinal disorders; CD3+=Cluster of differentiation 3+ T cells; CD4+=Cluster of differentiation 4+ T cells; MC=Mast cells; IBS-D=Irritable bowel syndrome with Diarrhea; IL2=Interleukin 2; IL8=Interleukin 8; IL10=Interleukin 10

Author	Year	Study design	Aim of study	Conclusions
Ivashkin et al. [22]	2021	Case-control	Evaluate the changes in gut microbiome and its components in IBS patients, and control patients as part of its pathogenicity.	Changes in the microbiome as result of persistent low-grade inflammation, impaired the gut permeability and exacerbate the IBS symptomatology.
Ellergezen et al. [23]	2021	Case-control	Evaluate the presence of immune mediators in the serum of patients with FMS and determine its relationship with the symptomatology.	There is a positive correlation between IL-17 and waking unrefreshed in FMS patients. There was a decrease of some pro inflammatory cytokines during the study, probably due to pregabalin used.
Khamisy-Farah et al. [5]	2021	Cross-sectional	Investigate the role of new inflammatory biomarkers as part of the diagnosis of FMS.	Compared with controls, there are higher CRP levels and PLR but lower lymphocyte count in the FMS group. FMS has inflammatory components may be useful in the its diagnosis.
O'Mahony et al. [27]	2021	Systematic Review	Discover the differences in cytokine levels in patients with FMS vs. HC.	There was a significant difference in the levels of cytokines in patients with FMS vs. HC. Nevertheless, these cytokines in blood were pro and anti-inflammatory.
Alciati et al. [29]	2021	Narrative	Summarize the updated information about FMS regarding diagnosis, pathogenesis, and treatment.	There is new information about the diagnosis, pathogenesis, and treatment of FMS that the physicians should be aware of to give the best options to these patients.
Erdrich et al. [8]	2020	Systematic Review	Identify the comorbidity of functional gastrointestinal disorders such as IBS in patients with FMS to discuss its implications.	There is a correlation between IBS in patients living with FMS, but there are more variations of FGIDs that could affect FMS patients. However, the data is not extensive to corroborate it.
Banfi et al. [6]	2020	Narrative	Provide an understanding of the role of immune cells in the physiopathology of FMS, especially T-cells, and the link between its symptoms.	There were non-conclusive results about the association of T cells in the maintenance of FMS symptoms. However, there is an alteration of the central pain pathways that produce increased inflammatory mediators, CD4+ cells abnormality, and some autoimmune response.
Zhao et al. [20]	2019	Cross-sectional	Examine the association among serum levels of monocyte chemotactic protein-1/chemokine C-C ligand 2 with disease severity of FMS.	Elevated serum levels of monocyte chemotactic protein1/chemokine C-C motif ligand two are linked to increased severity of FMS.
Krammer et al. [26]	2019	Systematic Review	Corroborate the role of MCs as a link between mucosal immune activity and the nervous system in producing IBS symptoms.	The results in this review supported the role of MC in the pathophysiology of IBS and are important in the frequency and severity of symptoms presented in IBS patients.
Lazaridis et al. [30]	2018	Narrative	Summarize the participation of an abnormal immune system, including immune cells and its mediators, in developing IBS.	There is an important role of innate immune response and low-grade inflammatory mediators in the system and intestinal mucosa in developing IBS.
İliaz et al. [18]	2018	Case-control	Evaluate immune cell infiltration in rectal and ileal mucosa in patients with IBS.	There were lower CD3+ and CD4+ cells in biopsy specimens in IBS patients, and there was no significant difference between IBS and HC in serotonin positivity and counts of intraepithelial lymphocytes.
Liu et al. [19]	2018	Case-control	Measure the leptin in diarrhea-predominant IBS and analyze its relationship with visceral sensitivity, MCs, and nerve fibers.	The increased levels of mucosal leptin interact with MCs and the nervous system to contribute to the pathogenesis of IBS-D. The results demonstrated significance.
Patel et al. [17]	2017	Case-control	Evaluation of the role of pro-inflammatory and anti-inflammatory cytokines in diagnosing IBS.	There was an increase in the levels of pro-inflammatory cytokines (IL2, IL8) and a reduction of anti-inflammatory IL10 in patients with IBS, which may have a role in its pathogenesis.
Yang et al. [4]	2017	Cohort	Examine the presence of diverse risk factors as part of the development of IBS and FMS.	During 11 years of follow-up, the incidence of IBS was higher in FMS patients, and after some adjustments in the study, the conclusion was that FMS increased the risk for IBS by 1.54-fold.

Microbiota and Gut Barrier Alteration

The surface of the intestinal mucosa epithelium has a characteristic microbiota in normal conditions. Still, when there is an overgrowth of bacteria or any pathogenic microorganism like viruses, parasites, or fungi, their products can produce an overactivation of the immune system if the pattern recognition receptors (PRRs) do not act properly or do not differentiate the normal over antigenic products.

The toll-like receptors (TLR) are part of the PRRs; the most common ones to be activated in response to any pathologic processes are TLR2, TLR4, TLR5, and TLR9. Their function in chronic inflammation is well known, as stated by Dlugoz et al., where the authors concluded that there is an upregulation in TLRs in the physiopathology of IBS due to their findings of the expression of TLR4, TLR5, and TLR9 in the small bowel mucosa of 23 patients with IBS [16].

We can support the fact that the alteration of the microbiome is an important factor in the development of symptoms in IBS, especially visceral hypersensitivity, based on the findings of Bednarska et al. They demonstrated that there is an increased passage of bacteria to the lamina propria in biopsies of patients with IBS, especially *E. coli and Salmonella.* This passage was enhanced due to the upregulation of vasoactive intestinal peptide (VIP), its receptors vasoactive intestinal peptide adenylate cyclase 1 (VPAC1), the high density of MCs, abnormal function of tight junctions in the intestinal mucosal cells, and overexpressed interaction with enteric glial cells (EGC), disrupting the normal function of the gut barrier and its permeability. Moreover, even a more significant conclusion is that when they blocked these receptors (VPAC1), they found a decreased passage of bacteria and clinical improvement of the symptomatology. This important feature demonstrates the role of the mast cells and their mediators and growth factors, such as nerve growth factors (NGF) in IBS [2,3,21,22]. 

Currently, there is not enough information about the alteration in the gut microbiota as part of the pathogenesis of FMS. Still, multiple studies described the involvement of the same connective tissue immune cells (MCs) and their inflammatory products in the development of FMS symptoms, specifically when these cells affect and stimulate the nervous system. Moreover, Erdrich et al. described an increased rate of IBS in subjects with other musculoskeletal conditions; thus, they support the probability of an interaction between the gut microbiota and the musculoskeletal system. Therefore, we suggest a deeper investigation regarding this subject. We summarize the information related to this subheading in Table 5 [8,29]. 

**Table 5:** Microbiota and gut barrier alteration mechanism involved in IBS and FMS IBS=Irritable Bowel Syndrome; FMS=Fibromyalgia syndrome; FGIDs=Functional gastrointestinal disorders; EGC=Enteric glial cells; MC=Mast cells; NGF=Nerve growth factor; IBS-D=Irritable bowel syndrome with Diarrhea; mRNA=Messenger RNA; TLR=Toll-like receptors; VIP=Vasoactive intestinal polypeptide

Author	Year	Study design	Aim of study	Conclusions
Ivashkin et al. [22]	2021	Case-control	Evaluate the changes in gut microbiome and its components in IBS patients, and control patients as part of its pathogenicity.	Changes in the microbiome as result of persistent low-grade inflammation, impaired the gut permeability and exacerbate the IBS symptomatology.
Alciati et al. [29]	2021	Narrative	Summarize the updated information about FMS regarding diagnosis, pathogenesis, and treatment.	There is new information about the diagnosis, pathogenesis, and treatment of FMS that the physicians should be aware of to give the best options to these patients.
Erdrich et al. [8]	2020	Systematic Review	Identify the comorbidity of functional gastrointestinal disorders such as IBS in patients with FMS to discuss its implications.	There is a correlation between IBS in patients living with FMS, but there are more variations of FGIDs that could affect FMS patients. However, the data is not extensive to corroborate it.
Meira de-Faria et al. [21]	2020	Case-control	Investigate the interaction between EGC and mast cells in IBS and how a gut-brain disorder is linked with increased intestinal permeability and mast cell activation.	There is an involvement of EGC and MC in the control of barrier function and a possible EGC-MC interaction that may alter IBS patients, producing permeability abnormality that contributes to the pathophysiology of IBS.
Xu et al. [3]	2017	Cross-sectional	Explore the role and interaction of nerve growth factors and mast cells in IBS pathophysiology.	The elevation of NGF in the mucosa and its interaction with mast cells and sensory nerve fibers may be a factor in the development of visceral hypersensitivity and impaired gut barrier function in IBS-D.
Dlugosz et al. [16]	2017	Case-control	Comparing the expression of specific toll-like receptors in patients with IBS vs. controls.	There is an increased mRNA expression of TLR such as 4, 5, and 9 in the mucosa of the jejunum of IBS patients due to the involvement of bacteria and abnormal immune response.
Bednarska et al. [2]	2017	Case-control	Determine the mast cell and vasoactive intestinal polypeptide (VIP) function in barrier regulation.	IBS patients had higher levels of VIP than controls, and the biopsies samples had higher levels of tryptase and larger numbers of mast cells.

Neurotransmitter and Nerve Fibers' Receptors' Disruption

In four of the analyzed articles, the role of the nervous system and the activation of nerve fibers due to receptors, genetic alterations, and mediators are discussed as part of the pathogenesis of these diseases. Mishima et al. talked about the dysfunction in the brain-gut-microbiome (BGM) as part of developing IBS with the intermediation of serotonin (5-HT) as a neurotransmitter. Serotonin influences GI motility, visceral hypersensitivity, mucosal inflammation, immune response, and brain function. The enterochromaffin cells (EC) are the cells in charge of secreting this product when inflammation occurs in the intestinal mucosa. The altered signaling and increased levels of 5-HT are one of the origins of the symptomatology of IBS, especially in IBS-D. The use of 5-HT inhibitors to treat IBS symptoms corroborates the information above. 

The authors demonstrated that the alteration of the microbiome with the increased presence of serotonin-producing bacteria such as *E. coli, Corynebacterium, Lactobacillus, Clostridium, Enterococcus*, and others, upregulated the signaling of 5-HT and its receptors (5-HT3 and 5-HT4) in nerve fibers of the gut and worsened the symptoms [25,31].

On the other hand, regarding FMS serotonin metabolism, Park et al. discovered a significative low level of 5-HT in blood samples and spinal fluids of patients with FMS. They found some polymorphism in genetic serotonin receptors, specifically 5-HT2A and serotonin transporter 5-HTT. They stated that the alteration in the 5-HTT gene produces a higher index of psychiatric symptoms such as depression, anxiety, and fatigue in patients with FMS. Nevertheless, they also explained that other authors did not find associations regarding this topic. Thus, the serotonin role in FMS might be a subject for future research. We summarize the information related to this subheading in Table 6 [7,19].

**Table 6:** Neurotransmitters and nerve fibers disruption mechanism in IBS and FMS presentation IBS=Irritable Bowel Syndrome; FMS=Fibromyalgia syndrome; GI=Gastrointestinal; 5-HIAA=5-hydroxyindoleacetic acid; HVA=homovanillic acid; IBS-D=Irritable bowel syndrome with Diarrhea

Author	Year	Study design	Aim of study	Conclusions
Mishima et al. [31]	2021	Narrative	Understand the microbiome and serotonin interaction in the pathogenesis of IBS.	Serotonin is an important neurotransmitter in the pathogenesis of IBS due to its function of increasing GI motility, pain, mucosal inflammation, and immune response activation.
Chojnacki et al. [25]	2018	Case-control	Evaluation of serotonin and dopamine secretion and metabolism in clinical subtypes of IBS.	Serotonin and dopamine secretion are altered in patients with IBS. 5-HIAA/HVA ratio may be a useful test for differentiating clinical types of IBS.
Liu et al. [19]	2018	Case-control	Measure the leptin in diarrhea-predominant IBS and analyze its relationship with visceral sensitivity, mast cells, and nerve fibers.	The increased levels of mucosal leptin interact with the nervous system to contribute to the pathogenesis of IBS-D. The results demonstrated significance.
Park et al. [7]	2017	Narrative	Evaluate the new information about the genes contributing to the development of FMS and its symptoms' severity.	Could be a genetic involvement in the development of FMS, especially polymorphisms in serotonin, dopamine, and catecholamine pathway genes that contribute to the severity of the symptoms.

Limitations

Our study had some limitations; one of them was the exclusion of studies not written in English that had well-researched information, as well as studies that explain the probability of a role of other cytokines and inflammation products found in animals and not studied yet in humans. In addition, there was a limited amount of studies explaining the role of serotonin and its metabolites as well as the role of microbiota in the pathogenic process of FMS. Few articles could describe the function of catecholamines in the severity of the symptoms in both diseases but their information was not conclusive. For all these limitations, further investigation is suggested in order to obtain a deeper understanding of these conditions and give the patients better treatment plans and quality of life.

## Conclusions

The diseases appraised in this systematic review have multifactorial etiologies, different associated factors, and evident symptoms and conditions presented in both diseases, such as fatigue, pain, hypersensitivity, depression, anxiety, and others, that could be correlated in a certain way. In this study, we discussed common processes involved in IBS and FMS. The most important ones are the abnormal immune system response, inflammatory pathway with innate immune cells activation, with special mention of mast cells and their mediators such as interleukins, chemokines, leptin, and others. In the same way, the altered secretion of a few hormones, neurotransmitters, and the upregulation of receptors localized in the intestinal mucosa and musculoskeletal system, can develop the symptomatology of these illnesses.

It is important to mention that the information provided about serotonin metabolism is not entirely elusive, but it is a promising correlation mechanism between these two diseases. We propose further investigation regarding serotonin regulation and how achieving a balance in the serotonin levels could positively affect the symptomatology of these diseases. The authors of the selected studies explained the participation of every substance and intermediary product in the pathogenesis of both syndromes broadly. Even though they did it separately, we needed to group all this information and explore the common processes and the correlation of these to prevent and have early management of IBS and FMS if they presented as comorbidity.
